# Microcystic Changes in the Retinal Internal Nuclear Layer Associated with Optic Atrophy: A Prospective Study

**DOI:** 10.1155/2014/395189

**Published:** 2014-02-23

**Authors:** Benjamin Wolff, Georges Azar, Vivien Vasseur, José-Alain Sahel, Catherine Vignal, Martine Mauget-Faÿsse

**Affiliations:** ^1^Rothschild Ophthalmologic Foundation, Professor Sahel Department, 25 rue Manin, 75019 Paris, France; ^2^Rothschild Ophthalmologic Foundation, Neuroophthalmology Department, 25 rue Manin, 75019 Paris, France

## Abstract

*Purpose*. This study aimed at assessing the prevalence of pathologies presenting retinal inner nuclear layer (RINL) microcystic perimacular changes associated with optic nerve atrophy (OA). The charts of patients presenting a significant defect of the Retinal Nerve Fiber Layer (RNFL) were included prospectively in this study. Patients were classified according to the etiology of the RNFL defect. Two hundred and one eyes of 138 patients were enrolled in this analysis. Retinal images obtained showed the typical hyporeflective perifoveal crescent-shaped lesion composed of small round hyporeflective microcysts confined to the RINL in 35.3% of the eyes. Those findings were found in 75% of eyes presenting hereditary OA, 50% of eyes presenting ischemic optic neuritis, 50% of eyes with drusen of the optic nerve (ON), 44.4% of eyes presenting a compressive OA, 32% of eyes presenting inflammatory optic neuropathy from multiple sclerosis, 18.5% of eyes presenting OA from undetermined origin, and 17.6% of eyes having primary open-angle glaucoma. This study demonstrates that microcystic changes in RINL are not specific to a disease but are found in OA of various etiologies. Moreover, their incidence was found to be dependent upon the cause of OA, with the highest incidence occurring in genetic OA.

## 1. Introduction

Optic nerve atrophy (OA) is a wide spectrum of hereditary or acquired optic neuropathies arising from various etiologies. The clinical characteristics of the disease include color vision deficits, loss of contrast sensitivity, scotomas of variable density with partial or total loss of visual acuity, and development of unilateral or bilateral atrophy of the optic nerve [1, 2].

High-resolution retinal imaging technologies, such as scanning laser ophthalmoscope infrared (SLO-IR) imaging and Spectral Domain Optical Coherence Tomography (SD-OCT), and both B-scans and “en face,” were shown to help define the location and extent of structural damage occurring in several chorioretinal diseases [3].

In a previous study [4], the authors had noticed the presence of macular microcysts in the retinal inner nuclear layer (RINL) in patients suffering from advanced OA. These microcysts are never observed in normal eyes.

In this study, using high-resolution retinal imaging technologies, the authors analyzed prospectively the incidence, in different ocular pathologies, of these RINL microcystic changes that are associated with atrophy of the optic nerve (ON).

## 2. Materials and Methods

### 2.1. Patients and Inclusion

This clinical study was conducted at the Department of Ophthalmology at Rothschild Foundation in Paris, France. The clinical charts of patients, who were known to have a significant defect of the Retinal Nerve Fiber Layer (RNFL) in at least one quadrant, as measured with SD-OCT, were included in this study. Exclusion criteria were the presence of any associated retinopathy such as diabetic retinopathy (DR), retinal vein occlusions (RVO), age-related macular degeneration (AMD), epiretinal membrane (ERM), viral retinitis, retinitis pigmentosa (RP), and radiation retinopathy. All patients who had previously had an intraocular surgery or taken any drug known to be toxic to the retina and/or the ON (e.g., Fingolimod or Sildenafil) were excluded as well. After an explanation of the purpose of the study and procedures to be used after the inclusion and during the followup, informed consent was obtained from all patients. The procedures used conformed to the tenets of the Declaration of Helsinki.

### 2.2. Examinations Performed

All patients with significant RNFL defect had had a detailed ocular and medical history, as well as a thorough bilateral ocular evaluation. The ocular examination included careful testing of standardized Early Treatment of Diabetic Retinopathy Study (ETDRS) visual acuity (VA), a thorough anterior segment examination, intraocular pressure (IOP) recording with a Goldmann applanation tonometer, and detailed fundus evaluation by indirect and direct ophthalmoscopy. In addition, all patients had 30-degree color fundus photographs centered on the macula and optic nerve, SLO-IR imaging, fluorescein fundus angiography (FFA), B-scans, and “en face” SD-OCT, to detect small round hyporeflective microcysts confined to the RINL.

### 2.3. “En Face” SD-OCT Analysis

Automated central macular thickness (CMT) was generated by an SD-OCT instrument. Using automated eye tracking and image alignment based on SLO images, the software allowed the averaging of a variable number of single images in real time (ART [Automated Real Time] Module; Heidelberg Engineering). Macular mapping consisted of 197 transverse sections in a 5.79 mm × 5.79 mm central retinal area. Tridimensional reconstruction generated by the pooling of these sections provided a virtual macular brick, through which 496 shifting sections in the coronal plane resulted in C-scan, or “en face” OCT, while B-scan, or conventional OCT, was derived from sagittal and transverse sections. The results were then compared with data from classical imaging, namely, fundus photography, FFA, and SLO-IR imaging. Retinal vessel crossing points were automatically used as constant landmarks to allow alignment of the “en face” SD-OCT images with that of the fundus SLO-IR imaging. Each layer was switched on and off to evaluate precisely the correspondence between the extent of lesions on “en face” SD-OCT and the adjacent layers. Average RNFL measurement and RNFL thickness in the temporal, inferior, nasal, and superior quadrants were obtained as well, using the same SD-OCT instrument.

### 2.4. Statistical Analysis

The data were entered into a personal computer and managed by a database program. Statistical analysis was performed using commercially available software (SPSS Version 20.0, Inc., Chicago, Illinois). Unpaired Student's *t*-tests were used for statistical comparison between CMT of involved eyes and that of control eyes. The statistical significance was set at *P* < 0.05.

## 3. Results 

Two hundred and one eyes of 138 patients [55 females (39.8%) and 83 males (60.2%), *P* = 0.231] demonstrating a significant RNFL defect in at least one quadrant (temporal, superior, inferior, or nasal) met the inclusion criteria and were included in the analysis. Mean patients age was 43.4 ± 3.3 years [range, 12–88 years]. Mean best-corrected visual acuity (BCVA) in the affected eye at the time of presentation was 20/30.  Table 1 summarizes the different etiologies responsible for the RNFL defect as shown in our patients.

Of the 201 eyes presenting those RNFL defects, there were 40 eyes (19.9%) presenting with hereditary optic atrophy [mitochondrial or autosomal dominant optic atrophy (ADOA)], 6 eyes (3%) with ischemic optic neuritis (ION), 4 eyes (2%) with drusen of the ON, 9 eyes (4.5%) with compressive OA as diagnosed with brain and orbital Magnetic Resonance Imaging (MRI), 27 eyes (13.4%) with inflammatory optic neuropathy from multiple sclerosis (MS), 27 patients (13.4%) with OA from undetermined origin, 85 eyes (42.3%) with primary open-angle glaucoma (POAG), 2 eyes (1%) with idiopathic intracranial hypertension (IIH), and 1 eye (0.5%) presenting with juxtapapillary toxoplasmic retinochoroiditis.

Further analysis of those eyes with RNFL defects with SLO-IR, B-scans, and “en face” SD-OCT showed in 71 eyes (35.3%) a hyporeflective perifoveal crescent-shaped lesion composed of small round hyporeflective microcysts confined to the RINL without any extension to the adjacent layers.

**Figure 1:**
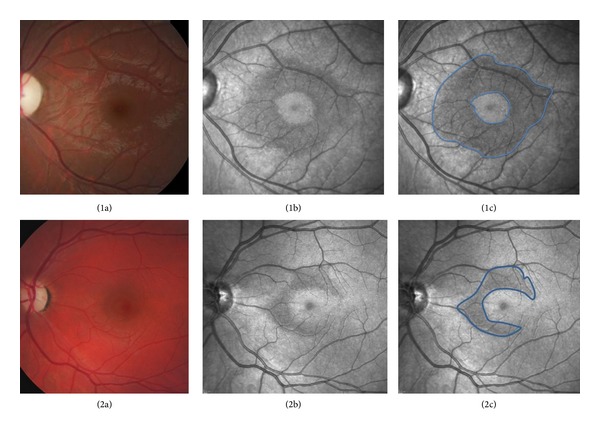
Color fundus photographs do not demonstrate retinal abnormalities (1a and 2a). RINL microcysts are detected with IR imaging (blue line delimitation) (1b, 1c, 2b and 2c).

**Figure 2:**
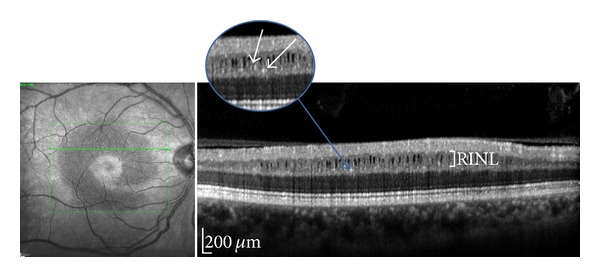
B-scan SD-OCT with magnification: hyperreflective pinpoint lesions observed within the RINL adjacent to the microcysts network in all cases (white arrows).

These microcysts measured between 20–30 microns to 70–90 microns within an *x*-*y* axis conjugate plane and were located between 500 and 2200 microns from the center of the fovea as measured with SD-OCT. The location of these cysts was variable. They could be found either superiorly, temporally, inferiorly, or nasally to the fovea (Figure 1). Associated hyperreflective pinpoint lesions were also observed within the RINL adjacent to the microcysts network in all cases (Figure 2).

**Figure 3:**
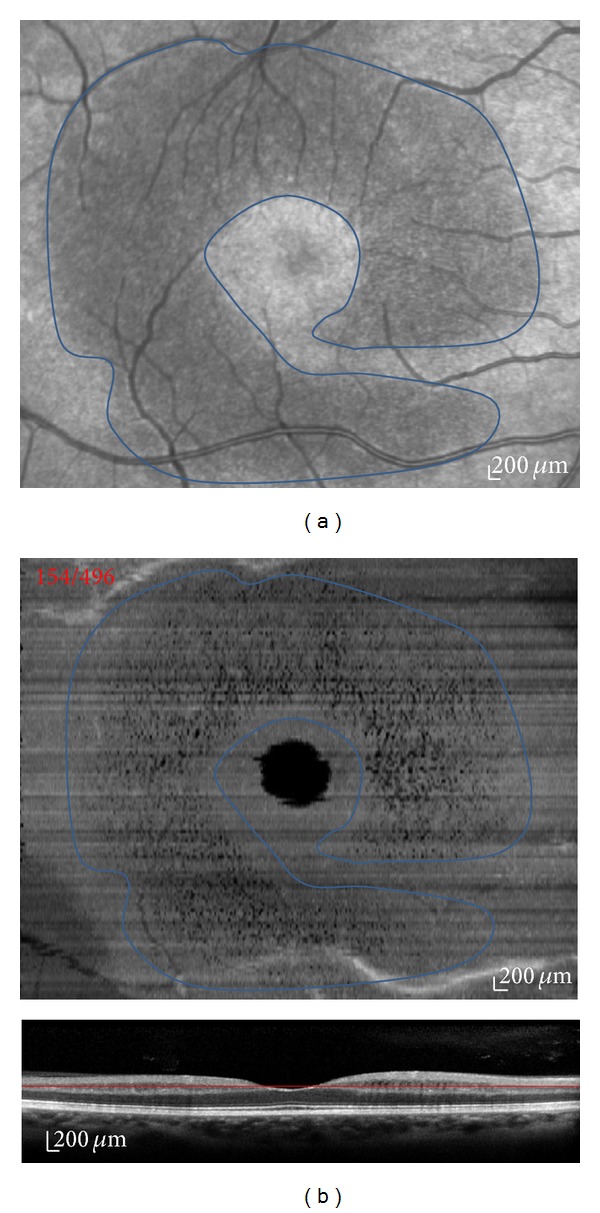
SLO-IR imaging (a): the crescent-shaped perimacular lesion correlated the with same hyporeflective area on the “en-face” SD-OCT (b).

As shown in Figure 3, this hyporeflective network lesion shown on the “en face” SD-OCT correlated with the same hyporeflective crescent-shaped perimacular lesion as shown with SLO-IR imaging. Mean age of patients presenting those microcysts was 44.13 years, whereas mean age was 58.40 in patients who did not.

As shown in Table 1, this round microcysts network was found in 30 cases (75%) of eyes presenting mitochondrial OA or ADOA, 3 cases (50%) of eyes presenting ischemic optic neuritis, 2 cases (50%) of eyes having drusen of the ON, 4 cases (44.4%) of eyes presenting a compressive OA, 12 cases (32%) of eyes presenting MS, 5 cases (18.5%) of eyes presenting OA from undetermined origin, and 15 cases (17.6%) of eyes having POAG. No similar lesion was found in eyes presenting IIH or toxoplasmic retinochoroiditis.

In all cases, the RNFL thickness of the involved eyes was significantly lower than that of the normal eyes when the etiology involved only one eye (Table 2). By stratifying our results to the different quadrants or clock hours around the optic disc, mean RNFL thickness in eyes presenting intraretinal cysts in the superior, temporal, inferior, and nasal quadrants were, respectively, 74.28 *μ*M, 35.28 *μ*M, 71.95 *μ*M, and 52.90 *μ*M. They were significantly lower than that of the fellow normal eyes, respectively, in the superior (113 *μ*M, *P* < 0.001), temporal (56.27 *μ*M, *P* < 0.001), inferior (115.90 *μ*M, *P* < 0.001), and nasal (65.54 *μ*M, *P* < 0.05) quadrants.

Mean central RNFL thickness of patients presenting OA was 58.50 *μ*M OD and 56.73 *μ*M OS when microcysts were present, and 62.90 *μ*M OD and 60.88 *μ*M OS when these were absent. No leakage was observed on fluorescein angiograms.

## 4. Discussion

This prospective study of a large population of patients with OA shows that microcystic changes in the RINL observed in OA are not specific of an etiology as previously thought in multiple sclerosis [5–8] but are found in many diseases of various etiologies, mostly genetic. Effectively, microcysts were found in 75% of eyes presenting mitochondrial OA or ADOA, 50% of eyes presenting ischemic optic neuritis, 50% of eyes having drusen of the ON, 44.4% of eyes presenting a compressive OA, 32% of eyes presenting MS, 18.5% of eyes presenting OA from undetermined origin, and 17.6% of eyes having POAG. No similar lesion was found in eyes presenting IIH or toxoplasmic retinochoroiditis.

The highest incidence found in patients with genetic OA could indicate that these microcysts may be directly linked to the dysfunction of the mitochondrial system rather than an inflammatory process. However, the exact underlying mechanism remains still unclear. It is known that the gene mutated in cases of hereditary OA such as Optic Atrophy Type 1 (OPA 1) is a gene that encodes a dynamin-related GTPase. Dynamin is a large GTPase regulating vesicular traffic and endocytosis at the plasma membrane that has been shown to maintain the mitochondrial genome [9]. On the other hand, it is known that Müller glial cells that are located within the RINL ensure the homeostasis of the retina by regulating neurotransmitters such as Glutamate and Gamma-Amino-Butyric Acid (GABA), thus protecting the adjacent retinal ganglion cells (RGCs) [10–13]. Therefore, we suggest that most of this mitochondrial dysfunction as well as accumulation of neurotransmitters could have led to pseudocysts in the RINL that could represent a microglial or Müller cells metabolism problem as seen with SD-OCT.

As the incidence of the RINL microcysts was different according to the pathology inducing OA, they may reflect a phase in the evolution of the disease. This sign must be taken into account for the presence of an OA due to an ocular or cerebral disease. Moreover recognizing these pseudocysts is crucial as they may be confused with cystoid macular edema.

During followup of already known OA, this sign must be looked for in order to appreciate the severity or evolution of the affection.

In conclusion, microcystic changes in RINL of patient with OA is a nonspecific finding and is easy to detect with new retinal imaging technologies, that should always be looked for. This clinical sign is frequently found in various diseases with OA and not only in MS.

Ultimately, as this study was based on a limited number of etiologies that had led to OA, further prospective studies with larger series as well as deeper molecular investigation remain mandatory in order to fully confirm our results and uncover the mysteries of the pathophysiology underlying these RINL microcysts.

## Figures and Tables

**Table 1:** 

Etiologies	Number of eyes	Eyes with cysts	Percentage
Hereditary optic atrophy (mitochondrial or autosomal dominant optic atrophy)	40	30	75%
Ischemic optic neuritis	6	3	50%
Drusen of Optic nerve	4	2	50%
Compressive OA	9	4	44.4%
Inflammatory optic neuropathy	27	12	32%
Undetermined origin OA	27	5	18.5%
Primary open angle glaucoma	85	15	17.6%
Idiopathic intracranial hypertension	2	0	0%
Juxtapapillary toxoplasmic retinochoroiditis	1	0	0%

**Table 2:** 

Optic nerve RNFL	RNFL with cyst	RNFL/normal	*P* value
Superior	74.28	113	*P* < 0.001
Inferior	71.95	115.90	*P* < 0.001
Nasal	52.90	65.54	*P* < 0.05
Temporal	35.28	56.27	*P* < 0.001

## References

[1] Johnston PB, Gaster RN, Smith VC, Tripathi RC (1979). A clinicopathologic study of autosomal dominant optic atrophy. *American Journal of Ophthalmology*.

[2] Votmba M, Fitzke V, Holder GE, Carter A, Bhattacharya SS, Moore AT (1998). Clinical features in affected individuals from 21 pedigrees with dominant optic atrophy. *Archives of Ophthalmology*.

[3] Wolff B, Matet A, Vasseur V, Sahel JA, Mauget-Faysse M (2012). En face OCT imaging for the diagnosis of outer retinal tubulations in age-related macular degeneration. *Journal of Ophthalmology*.

[4] Wolff B, Basdekidou C, Vasseur V, Mauget-Faysse M, Sahel JA, Vignal C (2013). Retinal inner nuclear layer microcystic changes in optic nerve atrophy: a novel Spectral-Domain OCT finding. *Retina*.

[5] Saidha S, Soltirchos ES, Ibrahim MA (2012). Microcystic macular oedema, thickness of the inner nuclear layer of the retina, and disease characteristics in multiple sclerosis: a retrospective study. *The Lancet Neurology*.

[6] Gelfand JM, Cree BA, Nolan R, Arnow S, Green AJ (2013). Microcystic inner nuclear layer abnormaities and neuromyelitis optica. *JAMA Neurology*.

[7] Green AJ, McQuaid S, Hauser SL, Allen IV, Lyness R (2010). Ocular pathology in multiple sclerosis: retinal atrophy and inflammation irrespective of disease duration. *Brain*.

[8] Gelfand JM, Nolan R, Schwartz DM, Graves J, Green AJ (2012). Microcystic macular oedema in multiple sclerosis is associated with disease severity. *Brain*.

[9] Pesch UEA, Fries JE, Bette S (2004). OPA1, the disease gene for autosomal dominant optic atrophy, is specifically expressed in ganglion cells and intrinsic neurons of the retina. *Investigative Ophthalmology and Visual Science*.

[10] Casson RJ (2006). Possible role of excitotoxicity in the pathogenesis of glaucoma. *Clinical and Experimental Ophthalmology*.

[11] Hare W, WoldeMussie E, Lai R (2001). Efficacy and safety of memantine, an NMDA-type open-channel blocker, for reduction of retinal injury associated with experimental glaucoma in rat and monkey. *Survey of Ophthalmology*.

[12] Hare WA, WoldeMussie E, Lai RK (2004). Efficacy and safety of memantine treatment for reduction of changes associated with experimental glaucoma in monkey, I: functional measures. *Investigative Ophthalmology and Visual Science*.

[13] Seki M, Lipton SA (2008). Targeting excitotoxic/free radical signaling pathways for therapeutic intervention in glaucoma. *Progress in Brain Research*.

